# Revealing the Roles of Part-of-Speech Taggers in Alzheimer Disease Detection: Scientific Discovery Using One-Intervention Causal Explanation

**DOI:** 10.2196/36590

**Published:** 2023-05-02

**Authors:** Bingyang Wen, Ning Wang, Koduvayur Subbalakshmi, Rajarathnam Chandramouli

**Affiliations:** 1 Department of Electrical and Computer Engineering Stevens Institute of Technology Hoboken, NJ United States; 2 Galois, Inc Arlington, VA United States

**Keywords:** explainable machine learning, Alzheimer disease, natural language processing, causal inference

## Abstract

**Background:**

Recently, rich computational methods that use deep learning or machine learning have been developed using linguistic biomarkers for the diagnosis of early-stage Alzheimer disease (AD). Moreover, some qualitative and quantitative studies have indicated that certain part-of-speech (PoS) features or tags could be good indicators of AD. However, there has not been a systematic attempt to discover the underlying relationships between PoS features and AD. Moreover, there has not been any attempt to quantify the relative importance of PoS features in detecting AD.

**Objective:**

Our goal was to disclose the underlying relationship between PoS features and AD, understand whether PoS features are useful in AD diagnosis, and explore which PoS features play a vital role in the diagnosis.

**Methods:**

The DementiaBank, containing 1049 transcripts from 208 patients with AD and 243 transcripts from 104 older control individuals, was used. A total of 27 PoS features were extracted from each record. Then, the relationship between AD and each of the PoS features was explored. A transformer-based deep learning model for AD prediction using PoS features was trained. Then, a global explainable artificial intelligence method was proposed and used to discover which PoS features were the most important in AD diagnosis using the transformer-based predictor. A global (model-level) feature importance measure was derived as a summary from the local (example-level) feature importance metric, which was obtained using the proposed causally aware counterfactual explanation method. The unique feature of this method is that it considers causal relations among PoS features and can, hence, preclude counterfactuals that are improbable and result in more reliable explanations.

**Results:**

The deep learning–based AD predictor achieved an accuracy of 92.2% and an *F*_1_-score of 0.955 when distinguishing patients with AD from healthy controls. The proposed explanation method identified 12 PoS features as being important for distinguishing patients with AD from healthy controls. Of these 12 features, 3 (25%) have been identified by other researchers in previous works in psychology and natural language processing. The remaining 75% (9/12) of PoS features have not been previously identified. We believe that this is an interesting finding that can be used in creating tests that might aid in the diagnosis of AD. Note that although our method is focused on PoS features, it should be possible to extend it to more types of features, perhaps even those derived from other biomarkers, such as syntactic features.

**Conclusions:**

The high classification accuracy of the proposed deep learner indicates that PoS features are strong clues in AD diagnosis. There are 12 PoS features that are strongly tied to AD, and because language is a noninvasive and potentially cheap method for detecting AD, this work shows some promising directions in this field.

## Introduction

### Background

Alzheimer disease (AD) is a serious and the most common form of dementia worldwide. In the United States, more than 5 million individuals are living with AD and AD-related dementia, which costed the nation US $244 billion in 2019. The National Academy of Sciences, National Plan to Address Alzheimer’s Disease, and Affordable Care Act through the Medicare Annual Wellness identify earlier detection of AD-related dementia as a core aim for improving brain health for millions of Americans.

Traditionally, brief cognitive screening tests and biological marker methods (usually neuroimaging [1-4] or cerebrospinal fluid examination [5]) have been used for identification. However, these approaches tend to be invasive, be expensive, and trigger patient compliance problems. Alternatively, spoken language is a rich and inexpensive source of information in the detection of cognitive status, even at the early stage.

Robinson et al [6] showed that patients with AD are more likely to have a reduction in vocabulary size and difficulty in correctly using verbs and nouns. Croisile et al [7] showed that patients with AD give a shorter speech, more implausible details, and syntactically simplified descriptions.

Recently, machine learning– or deep learning–based automated early-stage AD detection using linguistic features has been proposed and has demonstrated outstanding diagnosis accuracy. Eyigoz et al [8] demonstrated that a patient’s language performance in naturalistic probes can expose subtle early linguistic signs of progression to AD much before a clinical diagnosis of the impairment. Khodabakhsh et al [9] studied the diagnosis of AD using speech features extracted from a spontaneous conversation and obtained 90% AD detection accuracy. Machine learning– or deep learning–based methods allow for the use of latent features that go beyond handcrafted features and represent more sophisticated concepts. For example, word or sentence embeddings map words or sentences from a vocabulary to a vector of real numbers. Good embeddings will encode concepts similar to the adjacent vectors. Studies that used word embeddings for AD diagnosis include the studies by Karlekar et al [10], Wang et al [11], Palo and Parde [12], and Mahajan and Baths [13]. In addition to word embeddings, the study by Karlekar et al [10] used part-of-speech (PoS) features; the study by Wang et al [11] used PoS features and sentence embeddings; the study by Palo and Parde [12] used targeted psycholinguistic, sentiment, and demographic features; and the study by Mahajan and Baths [13] used recurrent neural networks to capture the temporal dynamics in speech recordings for improving the diagnosis accuracy.

However, most previous studies were performance oriented and constructed more complex models with an increasing number of features and modalities. Although these models achieve better diagnostic accuracy, they usually sacrifice transparency in the diagnosis-making process. This is because most of these complex models are deep learning based, which makes them inherently opaque, and not all the features are human interpretable. This is especially true if their influence on the prediction is not well understood. This opaqueness and lack of understanding of the contributions of individual features to the prediction has resulted in reluctance among the clinical community to use these methods in practice [14].

Explainable artificial intelligence (XAI) refers to methods that can reduce the opaqueness of deep learning models. XAI methods can be classified according to various criteria. One of the taxonomies is based on the format of explanation. Local or example-based explanation explains an individual prediction, whereas the global explanation explains the model behavior (eg, feature importance).

Beyond explaining the model’s internal mechanism, recent studies have used XAI methods for scientific discovery. XAI-based scientific discovery enables the discovery of insightful scientific concepts from model explanations obtained through XAI methods. Ginsburg et al [15] proposed Feature Importance in Nonlinear Embeddings for the analysis of cancer patterns in breast cancer tissue slides. Feature Importance in Nonlinear Embeddings automatically determines the important features that revealed previously unknown scientiﬁc attributes. Li et al [16] showed that concepts similar to Kepler laws of planetary motion and the Newton law of universal gravitation can be obtained through XAI methods.

### Objectives

Our goal was to disclose the underlying relationship between PoS features and AD. Ours is the first study to explore the predictive power of PoS features for AD diagnosis by using a well-performing transformer-based [17] model, which is trained to use PoS features for AD diagnosis. If a feature does not impact the decision of this predictor, then it stands to reason that this feature does not have much predictive power. Note that although PoS features were used in previous works for AD diagnosis, and impressive accuracies were achieved, they were usually combined with other features as inputs; hence, the effect of PoS features alone is unclear. In our study, we found that using only PoS features can still yield a high AD diagnosis performance with 92.2% accuracy. Hence, we believed that it would be interesting to discover which PoS features play vital roles in this prediction.

To understand the importance of any given feature for a particular problem, it is important to study the effect this feature has *globally* on all samples. To achieve this goal, we used an example-based explanation called counterfactual explanation (CFE) [18] on our predictor. Example-based explanation gives explanations for individual data samples. Then, we analyzed the statistical summary of the CFEs of a *group* of data samples to show the global effect of each input feature.

Conventionally, CFE aims to answer “Why” questions such as “Why the model’s decision is Y” or “What would have happened to Y, had I not done X?” The first step in obtaining CFE is to search for counterfactual examples, which are defined as the examples obtained by applying minimal changes to the features of the original example and having the predefined outputs. Then, CFEs can be extracted by comparing the differences between the original example and its counterfactual examples. For example, if the model’s prediction is changed from a patient with AD to healthy control as we manually increase the appearance of nouns by the minimal unit (eg, 1) in a data sample, then the CFE would indicate that the number of nouns used is an important factor for classifying the sample as data collected from a patient with AD.

However, when generating counterfactual examples, the conventional CFEs assume that features are *independent* of each other. This can result in counterfactual examples that are not feasible in the real world. For example, an infeasible CFE can suggest that the number of nouns decreased whereas the number of adjectives increased, which is anticausal because adjective words are usually used to modify noun words; hence, its appearance is supposed to increase or be unchanged as the number of noun words increases.

It is clear that conclusions drawn from potentially infeasible counterfactuals cannot be reliable. Hence, it is important to develop a causally away counterfactual intervention method for our purposes. We argue that the key to making the generated counterfactual examples feasible is ensuring that the generation process of counterfactual examples obeys causal rules. That is, as counterfactual examples are generated by making changes to some features, the causal consequences of these changes (eg, an increase in the number of nouns causes an increase in the number of adjectives) have to be considered.

To generate feasible counterfactual examples, we propose using a causal model that contains a directed graph that models the random variables by nodes and their causal relation by directed edges. Each edge in the causal model also encodes the causal function f: P→C, where C represents any variable that is modeled in the causal model, and P represents the variables that cause variable C. Then, one can generate counterfactual examples of the original example by performing interventions in the causal model. Performing interventions is the process in which some variables within a sample are changed to fixed values, and the rest of the variables are generated according to the causal functions (eg, f). A counterfactual sample can be regarded as a CFE if it can yield the predefined output.

To understand the importance of a single feature, we intervened on only one feature at a time for counterfactual generation. Hence, we named our proposed method one-intervention causal explanation (OICE). We then used the one-intervention causal examples to explain the importance of each feature by asking, “What would have happened to the output, had I intervened on feature A?” Moreover, using one intervention allowed us to systematically study the impact of the different features. Each feature (and its descendants) that is impacted by the parent feature in this one-intervention approach could be further analyzed using the structural causal model (SCM). Finally, we defined 3 metrics for quantifying the importance of the features in the decisions.

### Related Work: CFE Method

CFEs are a widely used method for generating explanations of a model’s decision and aim to answer “How the world would have to be different for a desirable outcome to occur” [18]. By studying these counterfactual instances, that is, by examining the difference between the original scenarios and the hypothesis or a possible suggestion about how the desired outcome can be obtained by changing some of the features, one can explain why a model arrives at a specific outcome. Generally, CFEs are generated by finding the minimal changes required to change the classification of this instance to the desired class. Wachter et al [18] formulated a general form for finding the CFEs *x^CF^*:



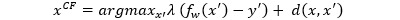



where *x* is the query instance, *f_w_* is the classifier, *y’* is the desired output, and *d (•,•)* is a distance function. In practice, maximization over *λ* is done by iteratively solving for *x’* and increasing *λ* until a sufficiently close solution is found.

The quality of CFEs is measured in terms of actionability, feasibility, diversity, and sparsity. The meaning of each metric is stated as follows:

- Actionability: refers to the extent a suggested alternative scenario or action is practical and feasible to implement. In contrast, a CFE that changes any immutable features (eg, gender: male → female) is unactionable.
- Feasibility: features that are changed by a CFE should be within a reasonable range or population. An infeasible CFE could be changing the number of credit cards from 5 to –1.
- Diversity: this refers to the ability to generate diverse CFEs.
- Sparsity: this refers to the number of features that are changed in CFEs. Fewer changes or high sparsity is favorable because humans can only extract limited information.

Most existing approaches in the literature on CFEs are dedicated to improving the aforementioned metrics. Recent studies [19,20] considered the distribution of data and generated counterfactual instances from the relatively high-density region of the input space. This method improves feasibility by avoiding unlikely or unrealistic counterfactual instances under the data distribution. Ustun et al [21] improved actionability and feasibility by allowing the counterfactual instances that optimize a user-specified cost function and prevent counterfactuals from changing immutable variables such as age, sex, and gender. Russell [22] proposed a mixed-integer programming formulation to handle mixed data types and offered CFEs for linear classifiers that respect the original data structure. This formulation is guaranteed to find coherent solutions by only searching within the “mixed-polytope” structure defined by a suitable choice of linear constraints.

The study most similar to ours is that of Karimi et al [23], which shifted the paradigm from the nearest CFEs to minimal interventions. Specifically, in the study by Karimi et al [23], counterfactual examples were generated by the predefined SCM and a set of possible interventions to achieve the desired outcomes. The optimal intervention set is obtained by choosing the optimal intervention set is the one that induces the minimum cost, where the cost is measured by a predefined cost function on the intervention sets. In addition, they proved the necessity of considering all intervariable causal dependencies and demonstrated efficiency on some toy data sets. We used a more complex SCM known as Causal Generative Neural Network (CGNN) [24] to capture the intervariable causal dependencies and generate CFEs using the intervention. In addition, we statistically analyzed the derived explanations to inspect the global behavior of the model.

## Methods

### Overview

**Figure 1:**
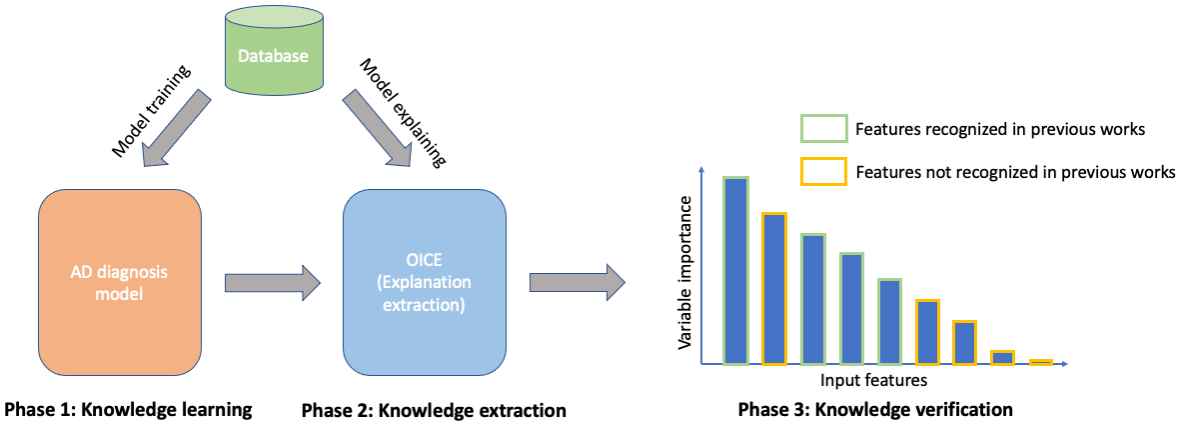
Method overview: procedures for using explainable artificial intelligence for scientific discovery. AD: Alzheimer disease; OICE: one-intervention causal explanation.

For scientific discovery purposes, our method incorporated 3 phases: knowledge learning, knowledge extraction, and knowledge verification. As shown in Figure 1, in the knowledge learning phase, we used a transformer-based classifier to learn the underlying association between PoS features and AD. In the knowledge extraction phase, we used our proposed XAI method, OICE, to extract the learned mechanism. In particular, OICE quantitatively indicated the importance of PoS features used by the model in AD classification, and the extracted knowledge (ie, feature importance) was verified with the findings of previous studies in phase 3. A model that is verified to have high consistency with previous findings is more plausible and, hence, is more likely to provide reliable insights into the underlying mechanism among PoS features and AD.

In the following sections, first, we introduce the data set and, subsequently, the structure of the transformer-based classifier. Then, we introduce the proposed model explanation method, OICE. Finally, we describe the details of implementing the introduced methods.

### Data Set Description

The DementiaBank [25] is a database of multimedia interactions for the study of communication in patients with dementia. This data set comprises the transcripts of individuals (individuals with dementia and control individuals) who were given four tasks: (1) cookie theft description, in which the participants in both the control group and dementia group were given a picture of a child attempting to steal a cookie and asked to describe what they saw; (2) word fluency, in which the fluency of the participants in the dementia group was measured; (3) recall, in which the participants in the dementia group were tested on their memory recall; and (4) sentence construction, in which the participants in the dementia group were tested on sentence construction. In total, the corpus contains 1049 transcripts from 208 patients with AD and 243 transcripts from 104 older control individuals, amounting to a total of 1292 transcripts. Two examples from the DementiaBank data set are presented in Table 1. In this study, we used all the transcripts described earlier.

The transcripts were tokenized into single-word tokens, and each token was computed with PoS tags using the Natural Language Toolkit [26]. Upon each transcript, we generated a PoS feature vector with the counts of 27 PoS tags. The names and the meanings of the 27 PoS features are presented in Table 2.

**Table 1:** Two examples from the DementiaBank data sample. In our experiment, we analyzed the part-of-speech features that were extracted from the speech records.

Label	Speech record
Healthy control	Okay, well the mother is drying the dishes, the sink is overflowing, um the little girl’s reaching for a cookie, and her brother’s taking cookies out of the cookie jar, and the stool is going to f knock him on the floor laughs, he’s going to fall on the floor because the stool’s not uh what, with gravity, whatever, uh the uh curtains are blowing I think, that’s all I can see
Patient with Alzheimer disease	I would like to have a lead pencil, the tree is blossoming, I hope my child doesn’t hafta go to the hospital, I hope my child doesn’t hafta go to the hospital, I shouldn't say that because we have a daughter who’s pregnant, and I do want her to go to the hospital, okay then, this winter has been a very cold one, the doctor said I, I sat in the chair by a the doctor, brief, I'm not, I forgot to try make them brief, the bureau drawer stands open

**Table 2:** Part-of-speech features and meanings.

Tag	Meaning
NN	Common noun
PRP	Personal pronoun
VBG	Verb in gerund or present participle form
UH	Interjection
NNS	Plural noun
MD	Modal verb
JJR	Comparative adjective
VB	Verb in base form
IN	Preposition or subordinating conjunction
JJ	Adjective
RP	Particle
PRP$	Possessive pronoun
CC	Coordinating conjunction
CD	Cardinal number
PDT	Predeterminer
NNP	Singular proper noun
TO	To
DT	Determiner
RB	Adverb
VBZ	Verb in third-person singular present form
VBN	Verb in past participle form
WP	Wh-pronoun
VBP	Verb in non–third person singular present form
JJS	Superlative adjective
VBD	Verb in past tense
EX	Existential there
WP$	Possessive wh-pronoun

### Ethical Considerations

We used the DementiaBank data set, which is archived by TalkBank. TalkBank is subject to its own Code of Ethics (detailed in the Code of Ethics page of the TalkBank website [27]), which supplements but does not replace the generally accepted professional codes of the American Psychological Association Code of Ethics and the American Anthropological Association Code of Ethics.

### Transformer-Based AD Classification Model

**Figure 2:**
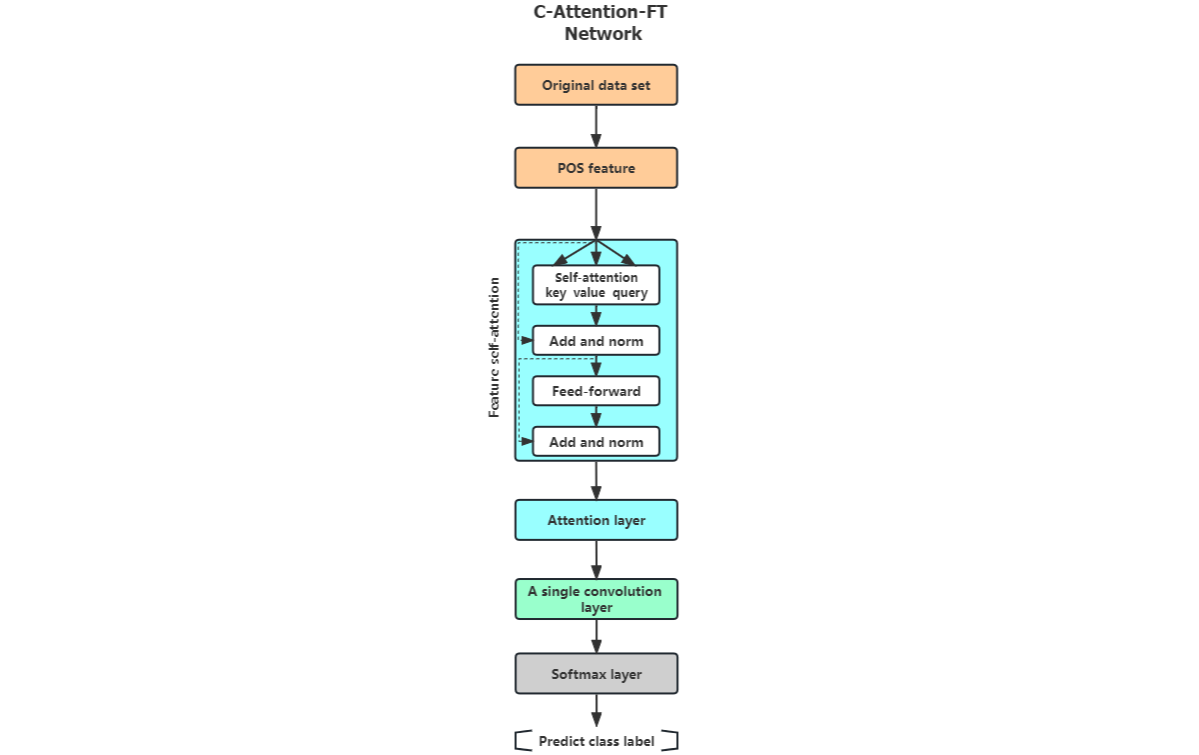
The proposed transformer-based classifier that uses the part-of-speech (PoS) features of the patient’s or control’s description. C: CNN; FT: feature.

Recently, we proposed a transformer-based [11] classifier to exploit PoS features, as shown in Figure 2. In our architecture, we used the multihead attention (MHA) module and the encoder structure of the transformer to process these features. Our motivation for this is stemmed from the success of this architecture in creating state-of-the-art language embeddings, as demonstrated in the study by Wang et al [11]. This architecture comprises a self-attention module that captures the intrafeature relationships, an attention layer together with a following 1-D Convolutional Neural Network layer. The MHA module is the same as that proposed in the study by Wang et al [11] for the popular transformer architecture. If *R = {r_1_, r_2,_ I, r_n_}* is the set of records, then *r_i_* is the *i*th record in the data set. We computed PoS features for each record. Let *P = {p_1_, p_2_, I, p_n_}* be the set of PoS feature vectors and *p_i_* be the *i*th vector in the PoS matrix. We used 6 MHA layers on *P = {p_1_, p_2_, I, p_n_}* to capture the relationship between the PoS features. The MHA transformed *P* into another matrix of *n*-dimensional vectors *A = {a_1_, a_2_, I, a_n_}*. The MHA module was followed by a 1-layer Convolutional Neural Network and a softmax layer to obtain the final classification.

### OICE Method

#### Overview

To derive an explanation, OICE first calculates the CFEs for each sample. Each CFE can simply be seen as a vote for the importance of the features of each sample. Then, OICE groups these CFEs to summarize the global explanation about feature importance. In this subsection, we first outline the preliminary information on SCM, which is an essential element for obtaining CFEs. We then describe how we learn an SCM from the data. Next, we discuss how we formulated OICE and how OICE generates individual CFEs using the pretrained SCM. Then, we introduce the metrics that we propose to measure feature importance (global explanation) according to a group of CFEs.

#### The Concept of SCM

In this section, we review the concepts of SCMs and interventions. An SCM, *M*, can be represented by a triplet, *M = (X, F, U)* that contains a set of endogenous variables, *X = {X_1_, X_2_, I, X_d_}*; a set of causal mechanisms, *F = {F_1_, F_2_, I, F_d_}*; and a set of exogenous variables, *U = {U_1_, U_2_, I, U_d_}*, where each *U_i_* is independently drawn from distribution *U*. Any endogenous variable *X_i_* can be obtained by its causal mechanism *F_i_* as *X_i_ = F_i_ (PA_i_, U_i_)*, where *U_i_ ~ U* and *PA_i_* denote the parent nodes of *X_i_* and *PA_i_ ε* *X* ***\*** *X_i_*.

In our case, the endogenous variables are the random variables of PoS features. The causal effect between 2 PoS features is, hence, encoded in the causal relationships between them (can be null if there is no causal relationship between them). The exogenous variables are seen as the set of unknown factors that can cause PoS features.

We denote an intervention in SCM by a do-operator *do (•)*. Intervening the set of *X* to the value *α* can then be described as *do ({X_i_ = a}_iεI_)* where *I* is a set of indices of the subset of endogenous variables to be intervened upon. By intervention, causal relations and causal mechanisms defined in the original SCM can be changed. Endogenous variables from *I* can be obtained through *do (X_i_ = a)* rather than *X_i_ = F_i_ (PA_i_, U_i_)*. Therefore, by performing the intervention, the original SCM *M* can be changed to a postintervention SCM *M_I_*.

#### SCM via Generative Network

**Figure 3:**
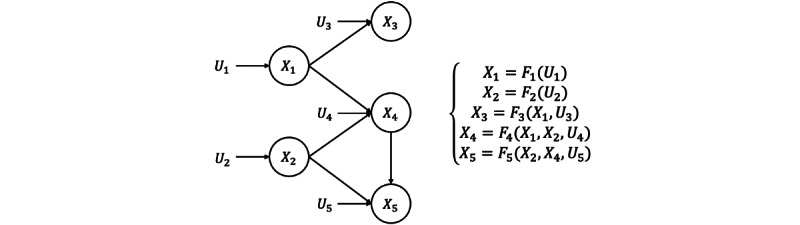
Example of a structural causal model. Left: causal graph. Right: causal mechanisms. X, U, and F stand for the endogenous variables, the exogenous variables, and the causal mechanisms, respectively. As for the Causal Generative Neural Network, each causal mechanism is implemented with a generative neural network.

We used the CGNN proposed in the study by Goudet et al [24] to represent SCM because it does not limit the types of causal mechanisms (eg, linear or nonlinear). Given a causal graph, a CGNN can be trained to learn the causal mechanisms underlying the causal graph by reducing the maximum mean discrepancy [28] between the ground-truth data and the generated data. CGNN generates each endogenous variable through *X_i_ = F_i_^θi^ (PA_i_, U_i_)*, where *F_i_^θi^* is a generative neural network parameterized by *θ_i_*. For simplicity, we use *F_i_* to represent *F_i_^θi^* in the rest of this paper. *U_i_* are random samples drawn from Gaussian distribution. Figure 3 illustrates an example of SCM construction using CGNN.

The weights of causal mechanisms (ie, *θ_i_*) are updated to minimize the maximum mean discrepancy between the ground-truth samples and the samples generated by the CGNN. In our experiment, we discovered the causal relations in the DementiaBank data set using the PC algorithm [29]. The PC algorithm is a constraint-based causal discovery method, under the assumption of causal sufficiency (ie, no latent confounders). We discovered causal relations among PoS features from the DementiaBank data set rather than using generic PoS causal rules, as the former would better capture the causal relations among PoS features in the dementia group.

#### Explanation by Minimal Intervention

We now introduce some notations and discuss the formulation of OICE. Let *x^F^ ε R^d^* denote the original factual sample and *x^CF^ ε R^d^* denote the counterfactual sample obtained by a set of interventions *I.* Here, we redefine *I = {I_1_, I_2_,…, I_d_}* to be an intervention set that has the same length as the sample *x^F^*. For each element *I_i_*, if *I_i_ = 0*, it denotes that no intervention was performed on *x_i_^F^* (the *i*th element of *x^F^*), otherwise it means that we performed the intervention *x_i_^F^ = I_i_*. Generally, any sample *x* (both factual and counterfactual) can be generated by SCM *(X, F, U)* using the equation: *x = G (U^F^, I; F)*, where G represents a sequence of processes to generate *x*. G contains a causal graph and the corresponding causal mechanisms between variables. The variables of a sample, *x*, are generated in sequence from the root to the leaf of the causal graph. The factual sample *x^F^* can be generated by setting all the elements in *I* to zero. For a given *x^F^*, its corresponding exogenous variable *U^F^* can be obtained by inverting the generating process: *U^F^ = G^–1^ (x^F^; F^–1^)*.

We formulated the problem of OICE as searching for the optimal I* that results in a counterfactual example *x^CF^*, which would flip the outcome from *y* to *y’.* One intervention was implemented by fixing the ||*I*||_0_ to be 1. It was formulated as follows:



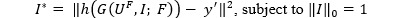



where *h* denotes the predictive model. In most cases, the model *h* is a probabilistic model; we then select the optimal solutions I* as those that result in counterfactual examples that can achieve a particular degree of certainty to be *y’* (eg, *h (G (U^F^, I; F)*) is 80% certain to be *y’*). Thus, multiple optimal solutions were obtained, which contain different intervened features. Note that the same type of intervened features may have different intervention values. Consequently, we further distilled our optimal solutions set by keeping only one solution for each subset with the same intervention that causes the minimum distance weighted by the median absolute deviation [18].

Note that OICE implicitly assumes the causal relation from variables *x^F^* to outcomes *y* by the predictive model *h*. However, OICE does not rely on this relation to generate counterfactual examples *x^CF^*. The model *h* in OICE only helps solve the optimization problem stated in Equation 1.

#### Metrics for Measuring Importance

So far, we have introduced how we obtained explanations for individual instances using OICE. We then made inference of the model’s global behavior (ie, importance of features) by statistically analyzing the explanations derived from a batch of samples. In this section, we introduce some metrics to measure the impact of intervening a feature to cause a flip in the outcome. The impact of features can be further associated with their importance for a machine learning model in making a decision.

Let *S = {S ^(1)^, S ^(2)^,…, S^(n)^}* represent a set of *n* samples that belong to class *y* (ie, *h (S^i^) = y, for I = 1, 2,…, n*). In our case, the problem is a binary classification problem, and the classes are “control” or “Alzheimer’s.” Let *C_k_^(i)^* denote the CFE of the *i*th sample obtained by intervening on the feature *k* and, hence, *h (C_k_^(i)^) ≠ y*. To measure the impact on the flip in the outcome caused by the intervening feature *k*, we introduce our first metric, *impact score (IS).* *IS_k_* can be interpreted as the proportion of counterfactual samples for which the feature *k* must be intervened to flip the outcome and is defined as follows:



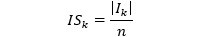



where *I_k_ = {i: h (C_k_^(i)^) ≠ y, i = 1, 2,…, n}* is a set that contains the indices of samples in *S* that have a CFE obtained by intervening on the feature *k*. The *IS* score describes the overall impact and does not consider the cost of the intervention (ie, how much a feature has been increased or decreased). Accordingly, we introduce another metric, *weighted IS (wIS),* to measure the impact made by changing the unit value of a feature. This measure trades off the impact with the cost of impact (CI). *wI*S can be used to draw comparisons among the features. Features with higher *wIS* values have more importance in flipping the outcome. To define *wIS*, we first introduce the parameter *CI* to measure the average absolute change that must be made to achieve the impact (ie, *IS*). Using subscript *j* to index the *j*th feature of a sample *S^(i)^* or *C_k_^(i)^* the *CI* for the feature *k* can be defined as follows:



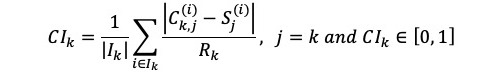



where *R_k_* is the range of feature *k*. Next, we define the *wIS* as follows:



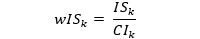



Note that the *wIS* defined in Equation 5 does not consider the trends of change in a feature (ie, increasing or decreasing). To address this, we separated *wIS_k_* into *wIS_k_^+^* and *wIS_k_^–^* to represent the *wIS* for increasing and decreasing the value of the feature *k*, respectively. They are calculated using the following rules: (1) if all the trends of change (ie, sign(*C_k,j_^(i)^ – S_j_^(i)^*)) are same, then *wIS_k_^δ^* is calculated using Equation 5, where *δ* is + if the changes are positive and − for negative; and (2) if both positive and negative changes exist, first, the *wIS_k_^+^* and *wIS_k_^–^* are calculated separately. Then the final *wIS_k_* is calculated by taking the average of the *wIS_k_^+^* and *wIS_k_^–^*. In addition, the IS introduced earlier measures the overall importance of changing both the intervened feature and its descendant features (caused by intervention on this feature).

It is important to understand how much each changed feature contributes to flipping the outcome. Consequently, we introduce another metric called *pure IS (PIS)* to quantify the importance of every changed feature within the CFEs obtained by the same intervention.

Hence, the *PIS* for a feature is calculated by subtracting the impact (on flipping the outcome) caused by its child nodes from the *IS* score of this feature. As the *wIS* represents the change in IS per unit change in the value of the feature, the impact of each child node is *m* and can, hence, be quantified as the average of the changes in *m*’s values multiplied by the *wIS* of *m*. The impact caused by the feature *m* when *m* is causally affected by the feature k is defined as follows:



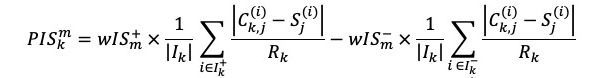



The *PIS* for the intervened feature *k, PIS_k_^k^*, is defined as follows:



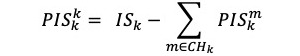



where *CH_k_* is the set of indices of the child nodes of the feature k. The value of *PIS_k_^m^* is then normalized over *IS_k_* to represent the percentage of effort for flipping the outcome.

### Implementation Details

#### Model Settings

In our experiments, we used 6 layers for the MHA module. We used stochastic gradient descent + momentum (SGD + Momentum) as the optimizer for training. Because DementiaBank is an unbalanced data set, we added a class weight correction by increasing the penalty for misclassifying the less frequent class during model training to reduce the effect of data bias. The class weight correction ratio used in this study is 7:3. We randomly split the original data into 81% training set, 9% validation set, and 10% testing set over multiple seeds. Our proposed model achieved a high accuracy of 92.2%, *F*_1_-score of 0.952, precision of 0.935, recall of 0.971, and area under the receiver operating characteristic curve of 0.971 on the DementiaBank data set.

#### PoS Feature Causal Relation Discovery

**Figure 4:**
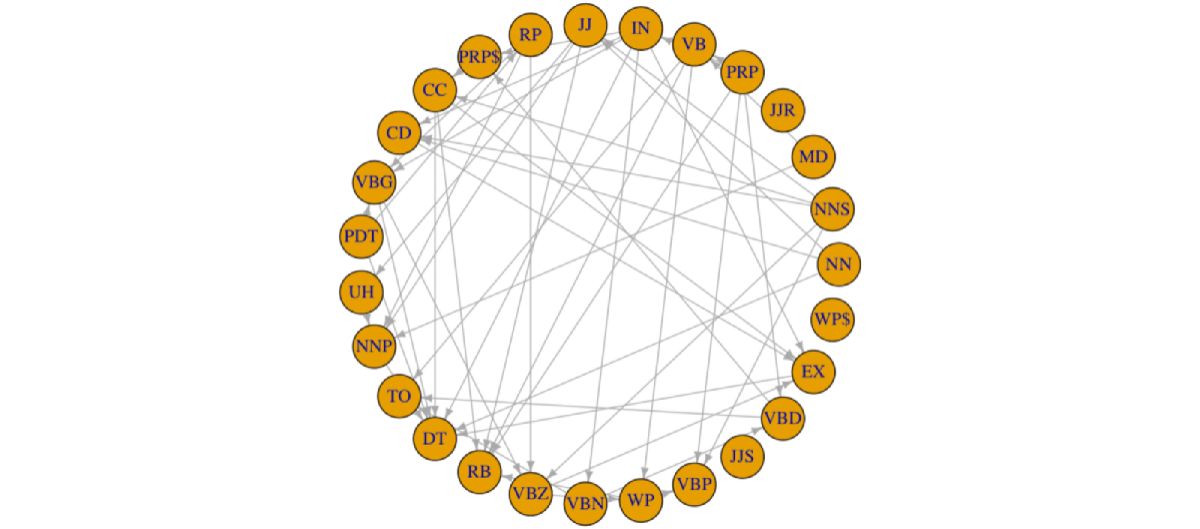
Causal graph for 27 linguistic features. The starting variable of each directed edge represents the cause, and the ending variable represents the effect. CC: coordinating conjunction; CD: cardinal number; DT: determiner; EX: existential there; IN: preposition or subordinating conjunction; JJ: adjective; JJR: comparative adjective; JJS: superlative adjective; MD: modal; NN: common noun; NNP: singular proper noun; NNS: plural noun; PDT: predeterminer; PRP: personal pronoun; PRP$: possessive pronoun; RB: adverb; RP: particles; TO: to; UH: interjection; VB: verb in base form; VBD: verb in past tense; VBG: verb in gerund or present participle form; VBN: verb in past participle form; VBP: verb in non–third person singular present form; VBZ: verb in third-person singular present form; WP: wh-pronoun; WP$: possessive wh-pronoun.

As mentioned earlier, we used the PC algorithm [29] to discover the intrafeature dependencies. The causal graphs returned by the PC algorithm contained undirected edges. Hence, we further revised the returned graph by orienting the undirected edges. The edges were oriented according to our knowledge of the linguistic features. For example, we made the causal direction noun (NN) → adjective (JJ) because NN causes the use of JJ. The full causal graph for the 27 linguistic features used in our experiment is illustrated in Figure 4.

#### Problem Solver

Solving the *l_0_* norm constraints in Equation 2 is a nontrivial task. However, the PoS features used by the proposed classifier are all integers and within narrow ranges. This makes it possible to solve the problem by exhausting all the solutions and then selecting the optimal ones. In addition, we set the certainty parameter to 80%; this implies that all solutions, *I*, that satisfy ||*h (G (U^{F}^, I; F)) – y’*||^2^ < *α*, where *α=.04*, are considered optimal. The value of *α* is chosen to reflect 80% certainty.

## Results

### Predictive Power of PoS Features

All PoS features described in Table 2 were used for model training. The model exhibits a high degree of performance, as evidenced by an accuracy rate of 92.2% and favorable precision, recall, and *F*_1_-scores of 0.935, 0.971, and 0.955, respectively, on the testing data set. In addition, the model yields a strong discriminatory capacity, as indicated by the Area under the receiver operating characteristic curve (AUC) of 0.971. The high performance illustrates that PoS features extracted from speech can help distinguish patients with AD from healthy controls. This finding encouraged us to move forward to explore which PoS features play a vital role.

### Knowledge Extracted From Model Explanation

In this section, we continue to reveal the important PoS features that direct the model’s decision. We analyzed the counterfactual examples from a statistical perspective and analyzed the important features derived from this analysis. We studied the CFEs for a control sample (ie, an individual without AD). The important features were derived by analyzing which feature plays a vital role in misclassifying a control sample as a patient with AD. In this paper, we report the results of 210 of the 243 controls. These 210 control samples were classified correctly by the classifier. The optimal CFE for all the 210 results could be achieved by intervening only one feature. Other samples were excluded because of misclassification.

**Figure 5:**
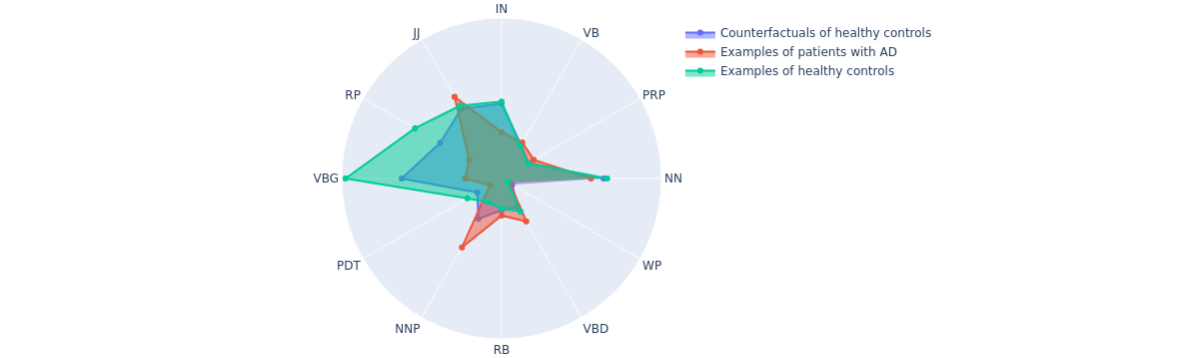
Spider plot for samples of patients with Alzheimer disease (AD), for those of healthy controls, and for counterfactuals of healthy controls (classified as those of patients with AD). IN: preposition or subordinating conjunction; JJ: adjective; NN: common noun; NNP: singular proper noun; PDT: predeterminer; PRP: personal pronoun; RB: adverb; RP: particles; VB: verb in base form; VBD: verb in past tense; VBG: verb in gerund or present participle form; WP: wh-pronoun.

We report both the *IS* and *wIS* for all PoS features in Tables 3 and 4. We regard the top 12 features (preposition or subordinating conjunction [IN], personal pronoun [PRP], particle [RP], verb in gerund or present participle form [VBG], predeterminer [PDT], singular proper noun [NNP], JJ, NN, verb in past tense [VBD], adverb [RB], verb in base form [VB], and wh-pronoun [WP]) as important PoS features in AD diagnosis. The selection considers PoS features that have both high *IS* scores and *wIS* scores. Features with low *IS* scores indicate that few samples adopt them for flipping the model’s output, which is less reliable owing to the lack of agreement by the majority. In Figure 5, we illustrate examples of patients with AD and healthy controls from the original data set and the counterfactual examples (explanation) in a spider plot. It shows that the generated counterfactual examples capture the difference in PoS features between patients with AD and healthy controls. The PoS features we used in this study are listed in Table 2. Further information on these features can be found in the Google Sites PoS tutorial [30] and the study by Toutanova et al [31].

**Figure 6:**
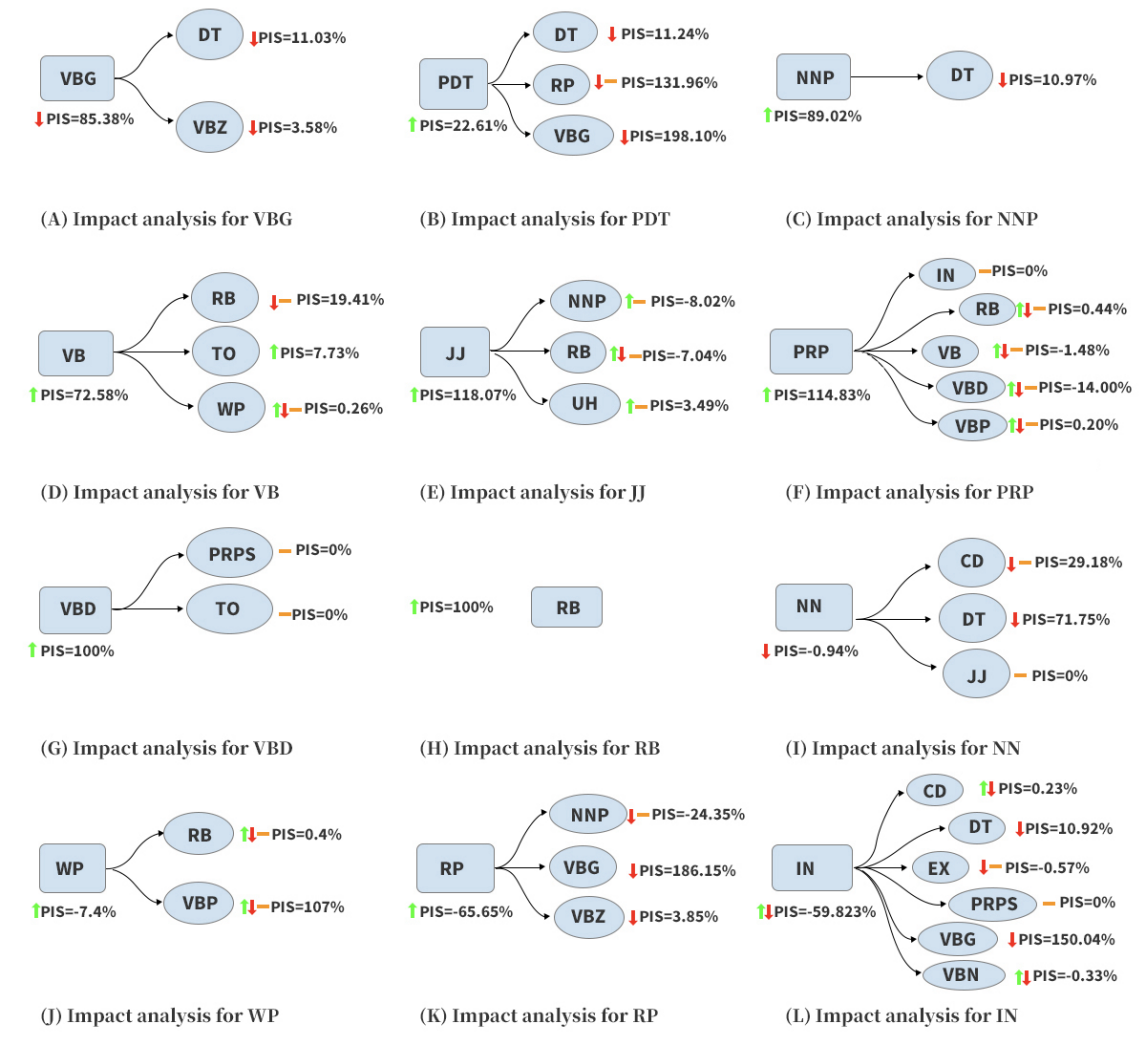
Explanations for the representative features. For an intervened feature, the red down arrow indicates that a decrease in value is required for flipping the outcome. For a child node (feature), the red down arrow indicates the change in direction caused by the intervention. The same rule applies to the green up arrow (an increase of value) and the orange horizontal line (no change of value). (A) Impact analysis for VBG; (B) Impact analysis for PDT; (C) Impact analysis for NNP; (D) Impact analysis for VB; (E) Impact analysis for JJ; (F) Impact analysis for PRP; (G) Impact analysis for VBD; (H) Impact analysis for RB; (I) Impact analysis for NN; (J) Impact analysis for WP; (K) Impact analysis for RP; (L) Impact analysis for IN. CD: cardinal number; DT: determiner; EX: existential there; IN: preposition or subordinating conjunction; JJ: adjective; NN: common nouns; NNP: singular proper noun; PDT: predeterminer; PIS: pure impact score; PRP: personal pronoun; PRPS: possessive pronoun; RB: adverb; RP: particles; TO: to; VB: verb in base form; VBD: verb in past tense; VBG: verb in gerund or present participle form; VBN: verb in past participle form; VBP: verb in non–third person singular present form; VBZ: verb in third-person singular present form; WP: wh-pronoun.

We then analyzed the important features to answer the following question: *how exactly does intervening a feature cause the outcome to flip?* To answer the above question, we considered the children features of the intervened feature given by SCM. More specifically, knowing that the counterfactual examples have moved across the decision boundary (ie, the outcome has flipped), we examined how each changed feature (ie, intervened features and their children) affects this movement of the original examples toward or away from the decision boundary. We used the normalized *PIS* (in terms of percentage) to quantify this effect. A positive *PIS* denotes the movement of the original examples toward the decision boundary and vice versa. In Figure 6, we illustrate how changes in each feature contribute to flipping the outcome and show 4 representative features as examples. First, we consider features to be “cooperative” (Figures 6A-6D) if both the intervened feature and its descendant features contribute to flipping the outcome. Second, we define the feature as “dominant” (Figures 6E-6H) if the intervened feature significantly contributes to flipping the outcome, while its descendant features make either no or an opposing contribution. Third, we classify the intervened feature as “idling” (Figures 6I and 6J) if it only slightly contributes to flipping the outcome, while the child features make a substantial contribution. Finally, we introduce the term “inverse” (Figures 6K and 6L) to describe a feature that moves the original instances away from the decision boundary upon intervention but causes other features to substantially push the original instances toward the decision boundary.

To complete the explanation that we promised at the beginning of this section, we use *CI* to quantitatively describe the average minimal changes that must be done to flip the outcome. In Table 5, we report CI and the changing direction (an up arrow means an increase in the value is required, whereas a down arrow means a decrease is required). For example, reducing the use of NN by 16.88% of the total range of NN feature will make the classifier flip the final decision.

Now, we combine the results from both Tables 4 and 5 to offer explanations for all important features. For clarity, in the following explanation, we do not use the words “increase,” “decrease,” and “change” to denote the actions that can modify the values of features. These 3 words are used to represent the pattern of how much the divergence of a feature from its real value can affect the decision of the model. We use “contribution” or “contribute” to denote the positive effort (measured by *PIS*) or process to flip the outcome. As opposed to “flip the outcome,” we use the terminology “consolidate the outcome” to denote that changing a feature causes the outcome to move further away from the decision boundary.

- *VBG*: decreasing the value of VBG by 20.7% causes the values of both determiner (DT) and verb in third-person singular present form (VBZ) to decrease. The decrements of VBG, DT, and VBZ contribute to flipping the outcome.
- *PDT*: increasing PDT by 20.2% causes VBG, DT, and RP to decrease or remain unchanged. VBG and DT contribute substantially to flip the outcome, whereas PDT makes only partial contributions.
- *NNP*: increasing NNP by 29.5% will cause DT to decrease. Increasing NNP contributes substantially to flipping the outcome, whereas the resulting decrements in DT make a partial contribution.
- *VB*: increasing VB by at least 69.3% will cause RB and WP to change or remain unchanged and cause “to” (TO) to increase. The changes in VB, RB, and TO contribute substantially to flip the outcome. The changes in WP make small contributions.
- *JJ*: increasing JJ by at least 16.5% will cause NNP and interjection (UH) to increase or remain unchanged and cause RB to change or remain unchanged. Even though the changes in NNP and RB consolidate the outcome, increasing JJ can substantially contribute to flipping the outcome. In addition, the change in UH makes a negligible contribution compared with the increment in JJ.
- *PRP*: increasing PRP by at least 18.1% will cause VB, IN, RB, verb in non–third person singular present form (VBP), and VBD to change or remain the same. However, by analyzing the *PIS* for the changes in these features, we conclude that PRP contributes substantially to flipping the outcome
- *VBD*: increasing VBD by at least 37.1% will not cause changes in PRP and TO. We conclude that VBD solely contributes to flipping the outcome.
- *RB*: RB does not have any descendants. We conclude that increasing RB by 49.6% will cause a flip in the outcome.
- *NN*: decreasing NN by 16.9% can cause cardinal number (CD), DT, and JJ to decrease or stay unchanged. Although the change in NN does not contribute to flipping the result, the resultant changes of CD and DT are sufficient to flip the outcome.
- *WP*: increasing WP by 67.1% can cause RB and VBP to increase, decrease, or remain unchanged. Although the changes in WP and RB do not contribute to flipping the result, the resultant changes in VBP are sufficient to flip the outcome.
- *RP*: increasing RP by 16.7% causes VBG and verb in third-person singular present form (VBZ) to decrease and NNP to either decrease or remain unchanged. The changes in RP consolidate the outcome. However, increasing RP can still flip the outcome because intervening on RP will cause the descendant features to change. These changes substantially contribute to flipping the outcome.
- *IN*: on average, either increasing or decreasing IN by 5.5% can cause CD, DT, existential there (EX), PRP, VBG, and verb in past participle form (VBN) to change or remain unchanged. Among all the descendants of IN, the changes in CD, EX, PRP, and VBN make negligible contributions to flipping the result. The change in IN consolidates the outcome, and the major contributions to flipping the outcome are influenced substantially by decreasing VBG and slightly by decreasing DT.

**Table 3:** Impact scores for the 27 part-of-speech features. Feature with a higher impact score value denotes more samples successfully flipping the model’s outcome by intervening on it.

Tag	Rank^a^	Meaning
NN^b^	12	0.457
PRP^c^	1	1
VBG^d^	4	0.948
UH^e^	21	0.024
NNS^f^	14	0.243
MD^g^	23	0.019
JJR^h^	25	0.010
VB^i^	9	0.762
IN^j^	1	1
JJ^k^	11	0.553
RP^l^	7	0.852
PRP$^m^	19	0.043
CC^n^	13	0.295
CD^o^	22	0.024
PDT^p^	5	0.914
NNP^q^	1	1
TO^r^	16	0.124
DT^s^	18	0.057
RB^t^	8	0.824
VBZ^u^	24	0.019
VBN^v^	17	0.067
WP^w^	10	0.581
VBP^x^	15	0.214
JJS^y^	26	0.010
VBD^z^	6	0.886
EX^aa^	20	0.038
WP$^ab^	27	0.005

^a^Ranking of the part-of-speech features based on their impact scores.

^b^NN: common noun.

^c^PRP: personal pronoun.

^d^VBG: verb in gerund or present participle form.

^e^UH: interjection.

^f^NNS: plural noun.

^g^MD: modal verb.

^h^JJR: comparative adjective.

^i^VB: verb in base form.

^j^IN: preposition or subordinating conjunction.

^k^JJ: adjective.

^l^RP: particle.

^m^PRP$: possessive pronoun.

^n^CC: coordinating conjunction.

^o^CD: cardinal number.

^p^PDT: predeterminer.

^q^NNP: singular proper noun.

^r^TO: to.

^s^DT: determiner.

^t^RB: adverb.

^u^VBZ: verb in third-person singular present form.

^v^VBN: verb in past participle form.

^w^WP: wh-pronoun.

^x^VBP: verb in non–third person singular present form.

^y^JJS: superlative adjective.

^z^VBD: verb in past tense.

^aa^EX: existential there.

^ab^WP$: possessive wh-pronoun.

**Table 4:** Weighted impact scores for the 27 part-of-speech features. Features with higher values denote more importance for machine learning model in making decisions.

Tag	Rank^a^	Meaning
NN^b^	8	2.71
PRP^c^	2	5.53
VBG^d^	4	4.59
UH^e^	22	0.05
NNS^f^	13	0.81
MD^g^	17	0.21
JJR^h^	25	0.01
VB^i^	11	1.1
IN^j^	1	14.52
JJ^k^	7	3.35
RP^l^	3	5.11
PRP$^m^	20	0.08
CC^n^	14	0.61
CD^o^	24	0.03
PDT^p^	5	4.52
NNP^q^	6	3.39
TO^r^	16	0.22
DT^s^	15	0.42
RB^t^	10	1.66
VBZ^u^	19	0.15
VBN^v^	20	0.08
WP^w^	12	0.87
VBP^x^	17	0.21
JJS^y^	25	0.01
VBD^z^	9	2.39
EX^aa^	22	0.05
WP$^ab^	27	0

^a^Ranking of the part-of-speech features based on their weighted impact scores.

^b^NN: common noun.

^c^PRP: personal pronoun.

^d^VBG: verb in gerund or present participle form.

^e^UH: interjection.

^f^NNS: plural noun.

^g^MD: modal verb.

^h^JJR: comparative adjective.

^i^VB: verb in base form.

^j^IN: preposition or subordinating conjunction.

^k^JJ: adjective.

^l^RP: particle.

^m^PRP$: possessive pronoun.

^n^CC: coordinating conjunction.

^o^CD: cardinal number.

^p^PDT: predeterminer.

^q^NNP: singular proper noun.

^r^TO: to.

^s^DT: determiner.

^t^RB: adverb.

^u^VBZ: verb in third-person singular present form.

^v^VBN: verb in past participle form.

^w^WP: wh-pronoun.

^x^VBP: verb in non–third person singular present form.

^y^JJS: superlative adjective.

^z^VBD: verb in past tense.

^aa^EX: existential there.

^ab^WP$: possessive wh-pronoun.

**Table 5:** Cost of impact (CI) in percentage and the direction of change for all 27 part-of-speech (PoS) features^a^.

PoS feature name	CI value (%)
NN^b^ ↓^c^	16.9
NNS^d^ ↑^e^	30
MD^f^ ↑	9
JJR^g^ ↑	83.3
PRP^h^ ↑	18.1
VB^i^ ↑	69.3
IN^j^ ↑↓	5.5
JJ^k^ ↑	16.5
RP^l^ ↑	16.7
PRP$^m^ ↑	30.3
CC^n^ ↑	48.7
CD^o^ ↑	75.6
VBG^p^ ↑	20.7
PDT^q^ ↑	20.2
UH^r^ ↑	33.3
NNP^s^ ↑	29.5
TO^t^ ↑	57.4
DT^u^ ↓	13.6
RB^v^ ↑	49.6
VBZ^w^ ↓	12.5
VBN^x^ ↑	86.7
WP^y^ ↑	67.1
VBP^z^ ↑	73.8
JJS^aa^ ↑	100
VBD^ab^ ↑	37.1
EX^ac^ ↑	67.2
WP$^ad^ ↑	100

^a^A smaller CI value denotes that smaller changes are needed.

^b^NN: common noun.

^c^The down arrow indicates decreasing the values.

^d^NNS: plural noun.

^e^The up arrow indicates increasing the values.

^f^MD: modal verb.

^g^JJR: comparative adjective.

^h^PRP$: possessive pronoun.

^i^VBG: verb in gerund or present participle form.

^j^IN: preposition or subordinating conjunction.

^k^JJ: adjective.

^l^RP: particle.

^m^PRP: personal pronoun.

^n^CC: coordinating conjunction.

^o^CD: cardinal number.

^p^VBD: verb in past tense.

^q^PDT: predeterminer.

^r^UH: interjection.

^s^NNP: singular proper noun.

^t^TO: to.

^u^DT: determiner.

^v^RB: adverb.

^w^VBZ: verb in third-person singular present form.

^x^VBN: verb in past participle form.

^y^WP: wh-pronoun.

^z^VBP: verb in non–third person singular present form.

^aa^JJS: superlative adjective.

^ab^VB: verb in base form.

^ac^EX: existential there.

^ad^WP$: possessive wh-pronoun.

## Discussion

### Principal Findings

First, the high performance of the AD diagnosis model on PoS features indicates that PoS features are rich clues of speech or language impairments that happen in patients with AD. Later, by explaining the model using our proposed OICE XAI method, we reveal several important linguistic biomarkers for early-stage AD detection. Some of the findings are consistent with the previous findings in psychology and natural language processing.

- RB is highly relevant to semantic impairment: the study by Varley [32] claims that RB shows a deictic purpose, which is more common in aphasics with a semantic impairment. Furthermore, in the study by Fraser et al [33], RB was proved to have higher correlations with a diagnosis of AD. Our one-intervention method shows that increasing the use of RB in the speech of a healthy control causes the same speech to be classified as that of a patient (from a control). Hence, our experiments align with previous findings that the increased use of RBs is an indicator of AD.
- Increased PRP use is an important sign of semantic dementia: the study by Almor et al [34] shows that patients with semantic dementia produced an increased number of PRPs than controls. The result is in line with our conclusion that increasing the number of PRPs in a control’s speech classifies it as a speech sample of a dementia patient.
- NN naming deficits indicate cognitive deficits: patients with AD show graceful degradation of using living and nonliving NNs [35]. We see the same decline in NN use when shifting from a control sample to a dementia sample.

The consistency between the findings of this study and those of previous studies implies that the model possibly learns useful clues about PoS features. It somewhat supports the point that the rest of the features that were not studied can offer new insights. To sum up, 3 of 12 important features (25%; RB, PRP, and NN) found by our method are consistent with previous findings. We also found 8 other important features that have not been reported yet, namely IN, RP, VBG, PDT, NNP, JJ, VBD, VB, and WP. Our work also seems to suggest that the most important feature may be IN or the use of prepositions. Further clinical studies may be necessary to verify this insight.

### Limitations and Further Study

For the scope of work considered here, we do not see any limitations; however, we do believe that there is good scope for further study in this area. More modalities can be used in designing an AD predictor. These modalities could include brain imagery and other traditional biomarkers. The OICE method can then be applied to all the features used to detect AD, leading to a much more nuanced understanding of the causal relations of these biomarkers. This could then lead to clinical trials that test these findings. A subset of noninvasive biomarkers may then emerge as important in predicting AD, which might, in turn, lead to easier-to-implement screens for the disease.

### Conclusions

In this study, we propose a novel CFE method called OICE to analyze the dominant linguistic features, specifically PoS features, that can be used for AD disease detection. We propose 3 metrics to evaluate the contributions of these features to the final decision of the model. We collected the explanations from the AD detection model of high accuracy and analyzed these explanations using the metrics we defined. The features declared as important in the detection of AD by our methods, namely RBs, pronouns, and NNs, are consistent with previous works in psychology and natural language processing. We also found a few other features that are important but have not yet been reported. Finally, by leveraging SCM, we further explained how these important features affect the decision-making process.

## Abbreviations

AD: Alzheimer disease
CFE: counterfactual explanation
CGNN: Causal Generative Neural Network
CI: cost of impact
DT: determiner
EX: existential there
IN: preposition or subordinating conjunction
IS: impact score
JJ: adjective
MHA: multihead attention
NN: common noun
NNP: singular proper noun
NNS: plural noun
OICE: one-intervention causal explanation
PDT: predeterminer
PIS: pure impact score
PoS: part-of-speech
PRP: personal pronoun
RB: adverb
RP: particle
SCM: structural causal model
TO: to
UH: interjection
VB: verb in base form
VBD: verb in past tense
VBG: verb in gerund or present participle form
VBN: verb in past participle form
VBP: verb in non–third person singular present form
VBZ: verb in third-person singular present form
wIS: weighted impact score
WP: wh-pronoun
XAI: explainable artificial intelligence

## Data Availability

The DementiaBank data set [25] used in this study is password protected and restricted to members of the DementiaBank consortium group. Accessibility to this data set can be granted after joining the DementiaBank consortium group as a member. For details about accessing the data set, please refer to the study by Boller and Becker [25].
